# Identification of a Potential Food Additive Mixture and Health Risk Assessment Using the Modified Reference Point Index (mRPI)

**DOI:** 10.3390/toxics14100929

**Published:** 2026-10-20

**Authors:** Veronika Karnel, Ulrike Mayerhofer, Antonia Griesbacher, Michael Tscherner, Johann Steinwider

**Affiliations:** 1 Department Risk Assessment, Austrian Agency for Health and Food Safety, Spargelfeldstraße 191, 1220 Vienna, Austria; 2 Department Statistic, Austrian Agency for Health and Food Safety, Zinzendorfgasse 27/1, 8010 Graz, Austria; 3 Department Data Management, Austrian Agency for Health and Food Safety, Spargelfeldstraße 191, 1220 Vienna, Austria

**Keywords:** food additive, mixture, cumulative risk assessment, modified reference point index, human health

## Abstract

Food additives (FAs) are intended to be used to improve the quality of food. Within the European Union, more than 300 FAs are currently authorized. Due to their intensive use, humans consume several different FAs daily. The amount and consumption of FAs themselves are influenced by their individual consumption patterns and brand loyalty. Although humans ingest mixtures of several FAs, the substances are still assessed on an individual basis. This study investigated potential cumulative health risks from FA mixtures using currently available toxicological, occurrence and food-consumption data. Forty-two relevant FAs and food additive groups were selected, as their estimated intake exceeded 50% of their corresponding acceptable daily intake (ADI) and they are authorized in relevant food categories. Toxicological data were screened for FAs, and their endpoints for liver, kidney, gastrointestinal, developmental toxicity, reproductive toxicity and hematotoxicity were identified. Based on the availability of toxicological and analytical data, a mixture of 18 FAs was considered. A potential health concern was identified for kidney toxicity (mRPI: 1.8–10.8, refined mRPI: 0.3–1.6) and developmental toxicity (mRPI: 13.6–56.1, refined mRPI: 0.9–3.1). The main risk drivers were steviol glycosides (E 960 a–d), contributing to 40% of nephrotoxicity risks and 18% of developmental toxicity risks, and glutamic acid—glutamates (E 620–625), contributing to 40% of developmental toxicity risks. The study demonstrates the applicability of the mRPI as a screening and prioritization tool for FA mixtures and highlights the need for improved occurrence and co-exposure data.

## 1. Introduction

Food additives (FAs) are used for various technological, sensory and nutritional purposes such as prolonging shelf-life, sweetening, modifying or stabilizing consistency, enhancing taste or color and preserving the color of the product. Due to their intensive use and diverse areas of application, billions of individuals ingest FAs on a daily basis. Estimates from a French study showed mean daily intakes of FAs of 113 mg/kg body weight (bw)/day (d) in children and 56.7 mg/kg bw/d in adults. A total of 125 FAs were identified as being consumed by at least one adult, while children consumed 122 different FAs. On average, 34% and 49% of the daily energy intake in adults and children, respectively, accounted for ultra-processed food, which also implies the intake of FAs [1]. Within the European Union, over 300 FAs are authorized under Regulation (EC) 1333/2008 [2]. The European Food Safety Authority (EFSA) regularly evaluates the toxicity of FAs as well as the dietary exposure based on scientific literature and use/occurrence data provided by industry or member states. However, the available data, especially regarding the use/occurrence, remain sparse. To fill this data gap, a European-wide monitoring will be implemented in 2027, with the aim of establishing a harmonized framework for member states to track real-world consumption levels of FAs.

FAs are highly regulated on an individual level; however, knowledge on health impacts of their cumulative intake and the potential of “cocktail effects” remains limited, not least because of lacking data on real-life exposure to food additive mixtures.

Only a handful of studies, primarily epidemiological studies, investigate the impact of FA mixtures on human health. Some studies indicated an association between FA mixtures and type 2 diabetes [3,4,5] and cardiovascular diseases [6] or potentially increased cancer risks [7,8]. A substantial body of literature is available for ultra-processed food, where FAs are widely and highly used. These studies, however, cannot be considered for evaluations of FAs, since other nutritional factors like energy density, fiber and micronutrient displacement may also have significant health impacts.

The identification of a real-life mixture is challenged, not only by food matrix effects and the patterns of co-consumption of food and beverages throughout the day but also by consumer behavior, particularly whether consumers remain brand loyal or not [1]. To collect data and close data gaps in a harmonized approach, the European Commission introduced the Recommendation (EU) 2023/965 on the methodology for the monitoring of food additives and food flavorings in May 2023. In this recommendation, food additives and food flavorings should be monitored at intervals of 10 or 15 years depending on their priority. Due to the virtually infinite number of possible mixtures and the limited occurrence data available to identify a real-life mixture, the prioritization process was implemented. FAs that are likely to occur in a mixture with relevant exposure levels are prioritized. In this study, only FAs with identified adverse health effects and derived acceptable daily intake (ADI) were considered.

This study aims to investigate the potential risks associated with mixtures of FAs based on currently available data. This implies the identification of common endpoints but also the evaluation of occurrence data. In addition, the applied approach was assessed for its ability to identify a realistic real-life mixture.

## 2. Materials and Methods

### 2.1. Identification of High-Priority Food Additives

To get an overview of all authorized FAs, a database was created, which was extracted from the corresponding Regulation (EC) No 1333/2008 Annex II Union list [2]. All relevant information, such as ADI, estimated dietary exposure, and the five food categories that contributed most to dietary exposure, was extracted from the corresponding EFSA scientific opinions. After building this database, FAs were prioritized according to European Commission recommendation (EU) 2023/965 on the methodology for monitoring food additive and food flavoring intake. Only substances of high priority were of interest for this study. A FA was of high priority if the dietary intake exceeded 50% of the ADI for any age group. In the next step, the identified high-priority FAs were checked for relevance with respect to the authorized food categories and the consumption patterns of the Austrian population. In general, if no EFSA scientific opinion was available for an FA, or if the FA was only used in food categories with low consumption, it was excluded from further selection. A total of 42 FAs were selected for further consideration in cumulative risk assessments, as given in Table 1.

### 2.2. Cumulative Assessment Groups

In line with the EFSA recommendations [9], cumulative assessment groups for the considered FAs were formed, based on common toxicological target organs. Toxicological endpoints indicated in the most recent scientific opinions published by the EFSA/Scientific Committee for Food (SCF) or in the manuscripts by the JECFA were considered.

Solely effects following oral administration (via diet, gavage, water or beverages) for which NOAELs/LOAELs/NOELs were derived by the EFSA, SCF and JECFA were considered. Local effects, effects that were not considered adverse or relevant by the EFSA Panel, effects considered not unambiguous (e.g., due to aging, physiological processes, etc.) as well as non-specific effects (e.g., due to severe toxicity) were not taken into account. In addition, no genotoxic and carcinogenic effects were considered in the course of data collection.

The following relevant organs/organ systems were identified: adrenal gland, brain, gastrointestinal tract, hematological system, heart, kidney, liver, lung, reproductive and developmental toxicity, spleen, thymus, thyroid and urinary bladder. Furthermore, general toxicity, comprising effects on body weight, body fat mass and composition as well as clinical signs (except developmental toxicity), was considered.

Table 2 provides an overview of the toxicological endpoints and the allocated FAs that were considered for this assessment. A detailed description of the endpoints is given in the Supplementary Material in Table S1.

### 2.3. Exposure Assessment

#### 2.3.1. Austrian Food Consumption Data

The latest Austrian food consumption data for adults (age 18–64 years) was published in 2017 [10]. Only participants (*n* = 2169) with two complete dietary records were included in the consumption data analysis. The food consumption data was FoodEx2 coded, a standardized food classification and description system created by the EFSA [11].

Specific recipes were identified to link with FA data at the recipe level rather than the individual food-item level. These included various soups, filled pasta products, pizza, croquettes, lasagne, and salad preparations (meat-based and fish-based varieties). Several individual food items (cake, mussels, shrimp and prawns) were reclassified to FoodEx2 level 3 to enable linkage with occurrence data, as occurrence data at more detailed levels were insufficiently represented.

For each participant, the mean consumption across both dietary survey days was calculated for each food item.

#### 2.3.2. Occurrence Data

The occurrence data originated partly from Austria and partly from the EFSA. Here, a public data request was made, and FA occurrence data from Belgium, Czechia, Denmark, France, Germany, Greece, Hungary, Ireland, Italy, Lithuania, Luxembourg and Slovakia from the years 2015–2025 were used. Only analytical data were considered for this exposure assessment. The occurrence data was FoodEx2 coded. FA concentrations given as exceeding a certain range threshold (e.g., >2000 µg/g) were excluded, along with various other food groups deemed irrelevant to the analysis (e.g., “N_A”, “A0EZJ”, “A0BY6”, “A0EYM”, “A0F0J”, “A0BXT”).

For each analyte and FoodEx2 code, a mean concentration was calculated across all available measurements. This calculation included all results assigned to the same FoodEx2 code or to more detailed codes nested within the hierarchical structure. A detailed description of FA occurrence data can be found in Table 3.

Since the occurrence data were left-censored, the lower bound (LB), medium bound (MB) and upper bound (UB) approach was used [12]. Occurrence data below the limit of quantification (LOQ) were set equal to zero (LB), to half of the LOQ (MB) and equal to the LOQ (UB).

#### 2.3.3. Exposure Estimation

Individual exposure was estimated for each participant by multiplying the mean FA concentration (µg/g) of each food item by the mean consumption amount (g/kg bw/d). The resulting value was divided by 1000 to express exposure in mg/kg bw/d. Weighted descriptive statistics were calculated for the empirical exposure distribution. Data weighting was stratified by sex and three age categories (18–<25 y, 25–<51 y, 51–<65 y).

To estimate the exposure the software R (version 4.6.0) was used.

### 2.4. Mixture Risk Assessment

To estimate the mixture risk, the modified Reference Point Index (mRPI) method was applied. Here, substance-specific uncertainty factors (UF) were derived by using the decision tree developed by Vejdovszky et al. [13]. and multiplied by the quotient of exposure and the reference point (RP) to obtain the Reference Point Quotient (RPQ) (Equation (1)). The sum of all RPQs in the mixture is the mRPI (Equation (2)). If the RP divided by the derived UF was lower than an existing health-based guidance value (HBGV) for the substance, the corresponding HBGV was considered instead. An mRPI above 1 indicates a potential health risk. To estimate the mRPI Excel (MS365) was used.

(1) $${\mathrm{RPQ}}_{i}=\frac{{\mathrm{Exposure}}_{i}*{\mathrm{UF}}_{i}}{{\mathrm{RP}}_{i}}$$

(2) $$\mathrm{mRPI}=\sum_{i=1}^{n}{\mathrm{RPQ}}_{i}$$

Additionally, the percent contribution of each substance to the mRPI was calculated to identify the main risk drivers (see Equation (3)).

(3) $$\mathrm{Contribution}=\frac{{\mathrm{RPQ}}_{i}*100}{\mathrm{mRPI}}$$

## 3. Results

For some FAs, no analytical data were available. Therefore, lycopene (E 160 d), ethyl lauroyl arginate (E 243), ß-cyclodextrin (E 459), carrageenan and processed eucheuma seaweed (E 407; E407 a), sucrose esters of fatty acids (E 473) and sorbitan esters (E 491–495) had to be excluded from this risk assessment.

For 18 FAs, analytical data were available; therefore, nine food colors, five preservatives, three sweeteners and one flavor enhancer were included in the cumulative risk assessment. Glutamic acid/glutamates (E 620–625), steviol glycosides (E 960 a–d), erythritol (E 968) and phosphates (E 338–343; E 450–452) were identified as the FAs with the highest exposure levels. A detailed description of the exposure estimation is given in Table 4.

### 3.1. Occurrence of the Chosen Food Additives on the Austrian Market

Since analytical data from Austria did not cover all selected FAs, data from other countries have been included. The Mintel database was used as a tool to see how the selected FAs are represented on the Austrian market. Based on this data query, which is shown in Table 5, it was also verified how many of the investigated FAs occur as a mixture in one product. There is no product on the Austrian market that contains all 18 FAs. The request in the Mintel database was further grouped by FAs and their corresponding food categories, and several combinations were tested. Ten products are on the market that contain steviol glycosides (E 960 a–d) and sorbic acid and its potassium salt (E 200–202) or E 960 a–d and phosphates (E 338–343; E 450–452), and three products were found that contained E 960 a–d and curcumin (E 100). Three products were on the market with erythritol (E 968) and E 338–343; E 450–452, and two with E 968 and E100. Glutamic acid/glutamates (E 620–625) occurred in combination with E 338–343; E 450–452 in 266 products and in combination with E 200–202 in 29 products. The following combinations also occurred on the Austrian market: E 620–625 and benzoic acid and sodium/potassium benzoate (E 210–213) (*n* = 8), E 620–625 and sulfur dioxide/sulfites (E 220–229) (*n* = 11), E 338–343; E 450–452 and E 220–229 (*n* = 34), E 338–343; E 450–452 and E 210–213 (*n* = 27), E 200–202 and E 210–213 (*n* = 207), E 338–343; E 450–452 and Potassium/sodium nitrite (E 249–250) (*n* = 754). One sample for each sweetener and E 220–229 is on the market. We were also able to identify triple combinations of FAs that occurred together in products in Austria as E 620–625, E 200–202 and E 249–250 (*n* = 15), E 620–625, E 338–343; E 450–452 and E 249–250 (*n* = 221), E 620–625, E 249–250, and E 210–213 (*n* = 3), E 200–202, E 210–213 and E 968 (*n* = 8), E 200–202, E 210–213 and E 960 a–d (*n* = 5), E 200–202, E 210–213 and aspartame (E 951) (*n* = 1) and E 338–343; E 450–452, E 220–229 and E 249–250 (*n* = 16).

### 3.2. Toxicological Endpoints

Cumulative assessment groups could be formed for toxic effects on the liver, kidneys, gastrointestinal tract, development, reproduction and hematology.

#### 3.2.1. Liver

Six FAs, four food colors and two preservatives were considered relevant for the liver endpoint. The effects on the liver reported for the food colors are described as histological changes/inflammation/lesions/hyperplasia. For preservatives, increased organ weight was described [14,15,16,17,18,19,20,21,22,23]. A more detailed description of the endpoints is given in the Supplementary Material Tables S1 and S2.

**Figure 1:**
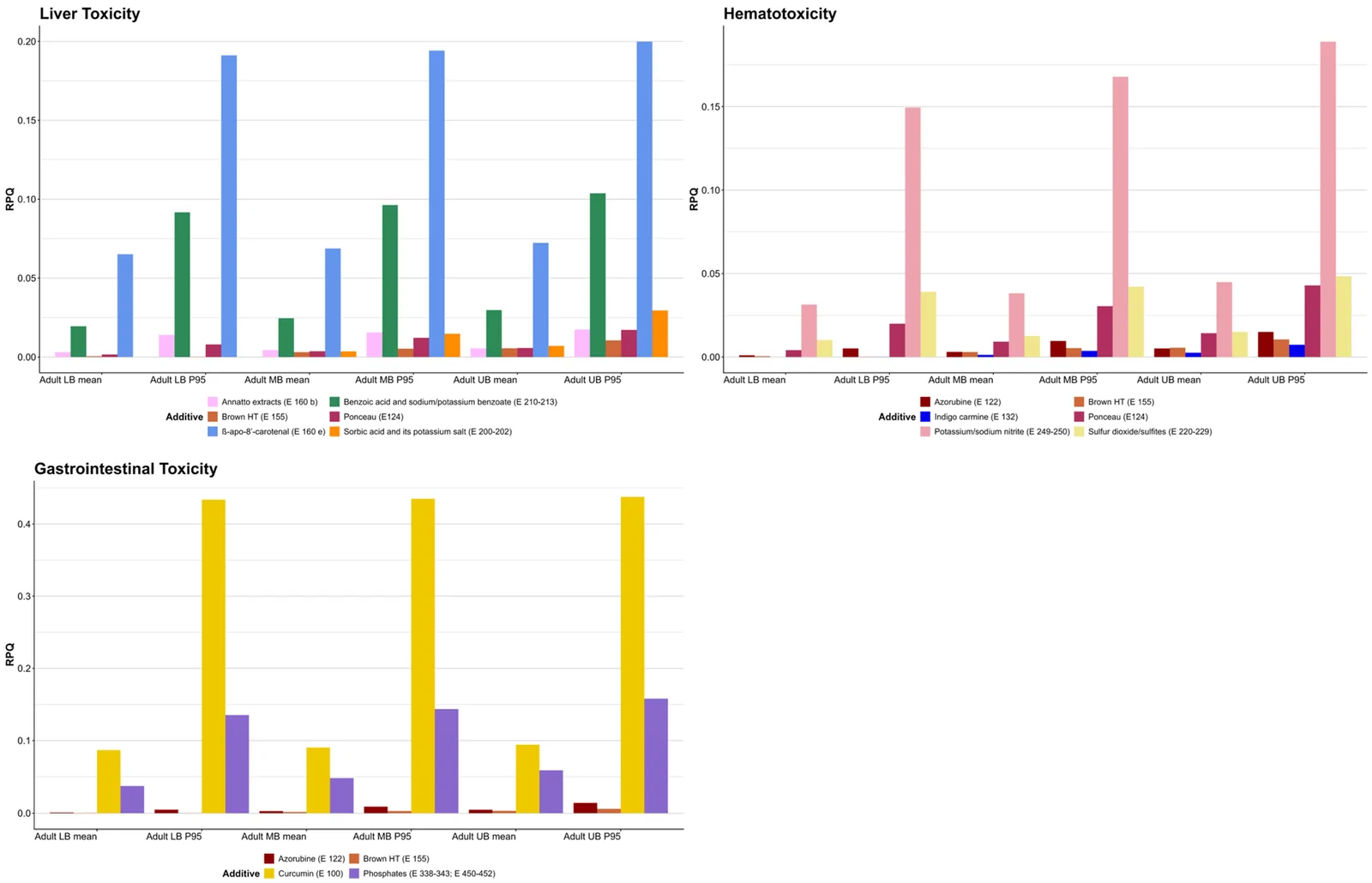
Overview of the RPQs of individual FAs in the cumulative assessment groups on liver toxicity, gastrointestinal toxicity and hematotoxicity. The RPQs are given as mean and 95th percentile in lower, medium and upper bounds. The sum of the RPQs in each group did not exceed the mRPI threshold of 1.

The mRPI ranged between 0.09–0.13 at mean and 0.30–0.38 at high exposure (95P), from lower bound to upper bound. Since the value is below 1, potential health risks regarding liver toxicity associated with this mixture are considered unlikely. In Figure 1, the individual RPQs of each FA are shown. The preservatives sorbic acid (E 200–202) and benzoic acid (E 210–213) were identified as the main risk contributors for this endpoint, with an average contribution of 61% and 26%, respectively.

#### 3.2.2. Gastrointestinal Toxicity

For this cumulative assessment group, a total of four FAs, three food colors and one preservative were considered relevant. The effects of these substances on the gastrointestinal tract were described as histological changes [15,16,24,25,26,27,28]. A more detailed description of the endpoints is given in the Supplementary Material Tables S1 and S3. The mRPI ranged between 0.13–0.16 (LB-UB) and 0.57–0.62 (LB-UB) for mean and high exposure (95P.), respectively. Since the value is below 1, potential health risks regarding gastrointestinal toxicity associated with this mixture are considered unlikely. The individual RPQs are given in Figure 1. The main contributor to this endpoint is curcumin (E 100) followed by phosphates (E 338–343; E 450–452) with an average contribution of 69% and 29%, respectively.

#### 3.2.3. Hematotoxicity

Six FAs were considered relevant for this endpoint, consisting of four food colors and two preservatives. The described effects on the hematological system were alterations in hematological parameters and formation of methemoglobin [14,15,16,26,29,30,31].

A more detailed description of the endpoints is given in the Supplementary Material Tables S1 and S4. The mRPI for the mean exposure ranged between 0.05 and 0.09 (LB-UB), and the high exposure (95P) ranged between 0.21 and 0.31 (LB-UB). Since the value is below 1, potential risks of hematotoxic effects associated with this mixture are considered unlikely. The individual RPQs are shown in Figure 1. The main contributor, with an average contribution of 62%, was potassium/sodium nitrite (E 249–250), followed by sulfur dioxide/sulfites (E 220–229) with 18%.

#### 3.2.4. Kidney

In total, eleven FAs, four food colors, four preservatives and three sweeteners were considered relevant regarding adverse effects on the kidneys. The effects of these substances on the kidneys are described as histological changes and changes in relevant kidney parameters in the plasma [14,15,16,17,18,19,20,21,27,28,30,31,32,33,34]. The mRPI for the mean exposure scenario was 1.8–1.9 (LB-UB), and for the high exposure (95P) it was 10.5–10.8 (LB–UB). Since the value is above 1, potential risks of nephrotoxicity in association with this mixture cannot be excluded. The main contributors for this endpoint are steviol glycosides (E 960 a–d), with an average contribution of 95%.

**Figure 2:**
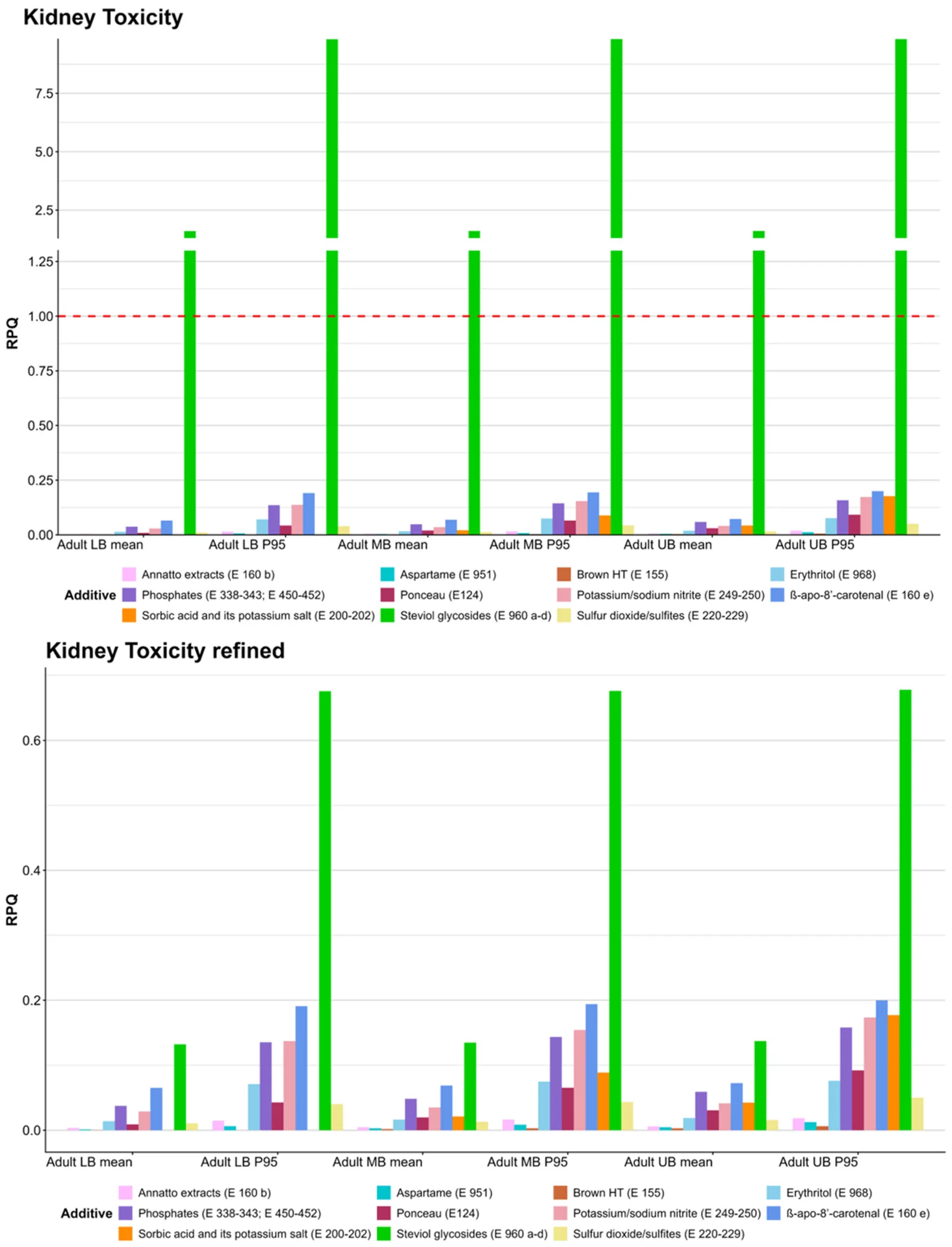
Overview of the RPQs of the FAs that have been identified for the kidney endpoint. The red dotted line is the threshold for the mRPI, which is only exceeded through the contribution of steviol glycosides (E 960 a–d).

The occurrence data from steviol glycosides (E 960 a–d) showed quite high levels in a small number of samples. Four tea samples with exceptionally high stevia concentrations were suspected to represent measurements of tea leaves rather than tea extracts and were therefore excluded from further analysis. The revised exposure estimation is given in Table 6. The kidney endpoint was recalculated with the refined exposure for steviol glycosides (E 960 a–d), and the mRPI was reduced to 0.3 to 1.6 (mean LB–P95 UB). The individual RPQs of each FA with the original exposure scenario, but also with the refined exposure scenario, are shown in Figure 2. A more detailed description of the endpoints is given in the Supplementary Material Tables S1, S5 and S6.

#### 3.2.5. Reproduction Toxicity

Four FAs were identified for this endpoint, including three food colors and one sweetener. The reproductive effects of these substances were described as increased uterus weight, increased cystic ovaries or decreased absolute weight of ovaries and decreased reproductive success [15,16,33,35,36,37]. The mRPI for the mean exposure was 1.6 (LB-UB), and for the high exposure (95P) was 9.7 (LB–UB). The main contributor, with an average contribution of 99.37%, was steviol glycosides (E 960 a–d). Here again, a refined exposure assessment, excluding the above-mentioned tea samples, was performed, and the risk was reduced to 0.14 to 0.73 (mean LB-95P UB).

**Figure 3:**
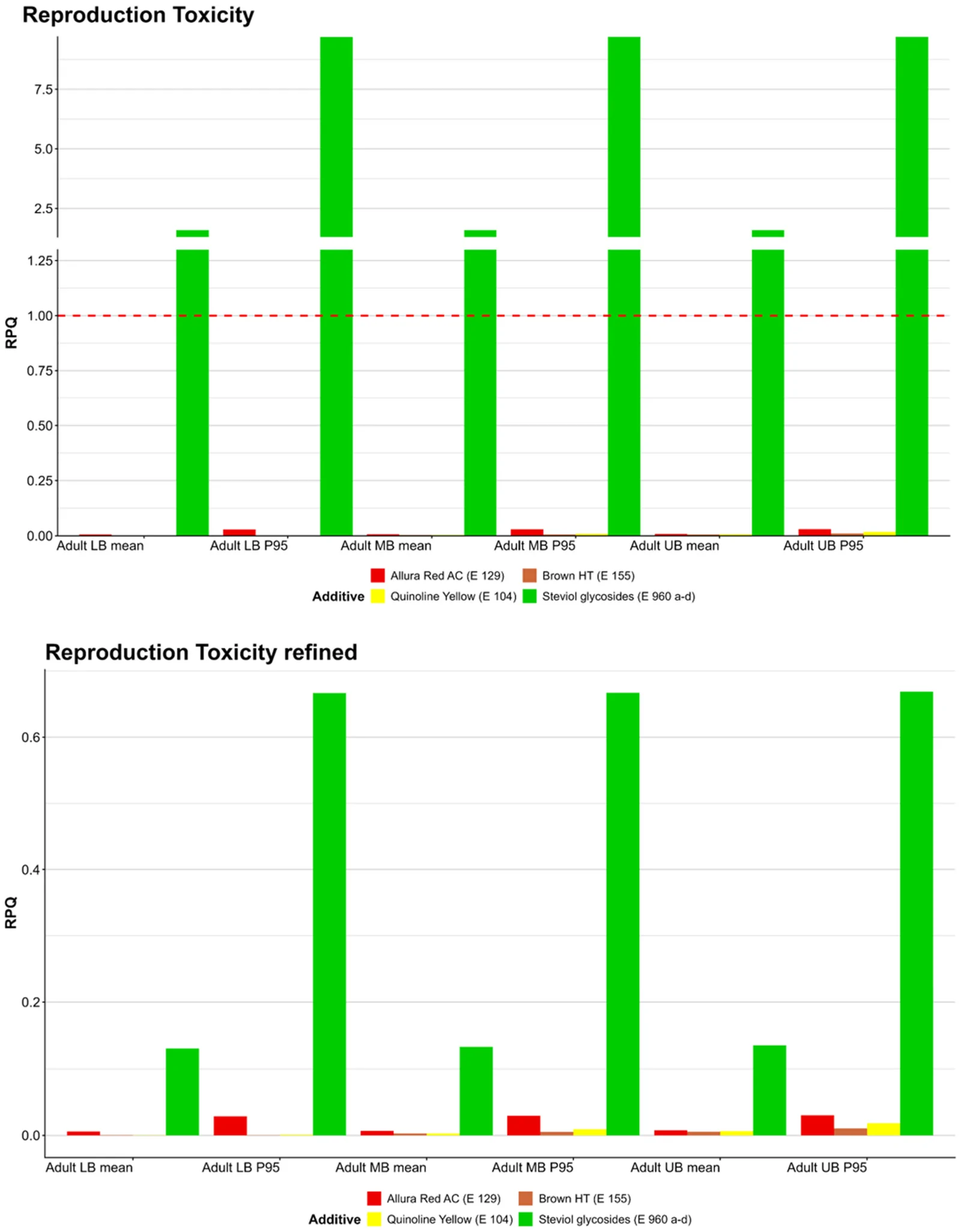
Overview of the RPQs of the FAs that have been identified for the reproduction endpoint. The red dotted line is the threshold for the mRPI, which is only exceeded through the contribution of steviol glycosides (E 960 a–d).

The individual RPQs of each FA with the original exposure estimation, but also with the refined exposure estimation, are shown in Figure 3. A more detailed description of the endpoints is given in the Supplementary Material Tables S1, S7 and S8.

#### 3.2.6. Developmental Toxicity

Nine FAs were considered for this endpoint: four food colors, two preservatives, one flavor enhancer and two sweeteners. The developmental effects described for these substances were mainly decreased body weight gain of the offspring, but also reduced ossification of the hyoid in the fetus, malformations, increased mortality and lower number of fetuses per litter or neurobehavioral effects [14,21,22,23,24,25,32,33,35,36,37,38].

The mRPI for the mean exposure was 13.6 (LB-UB), and for the high exposure (95P) it was 56 (LB-UB). The main contributors were glutamic acid/glutamates (E 620–625) and steviol glycosides (E 960 a–d), with an average contribution of 83% and 15%, respectively.

**Figure 4:**
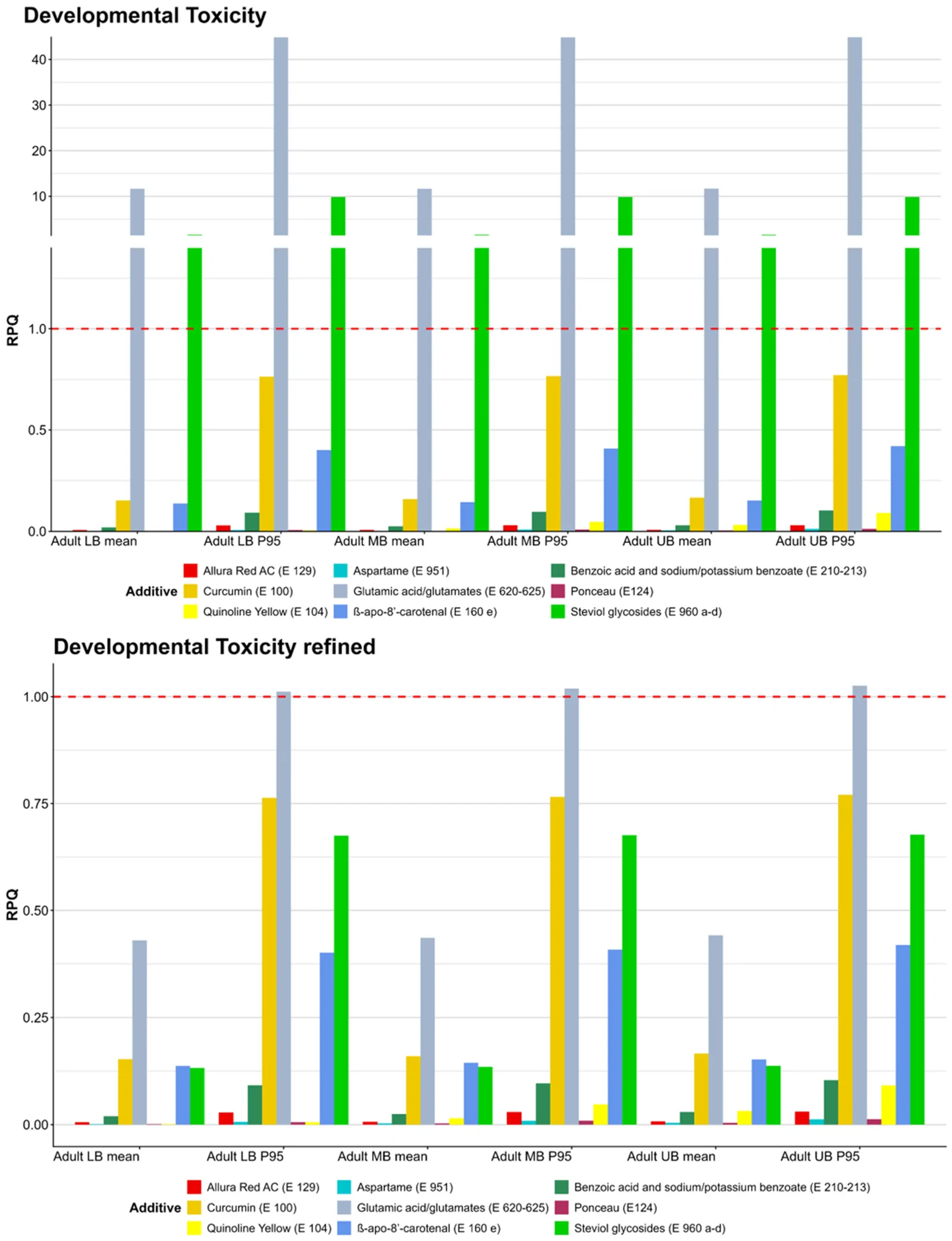
Overview of the RPQs of the FAs that have been identified for the developmental toxicity endpoint. The red dotted line is the threshold for the mRPI, which is only exceeded through the contribution of glutamic acid/glutamates (E 620–625) and steviol glycosides (E 960 a–d). In the refined exposure scenario, only E 620–625 slightly exceed the mRPI threshold of 1.

In the occurrence data from glutamic acid/glutamates (E 620–625) and steviol glycosides (E 960 a–d), quite high levels were detected, but only in a small number of samples. For glutamate, several samples were excluded based on the assumption that it reflects the natural occurrence of glutamate more than its use as a FA. As mentioned above for E 960 a–d, four tea samples were excluded. The revised exposure estimation is given in Table 5. Due to the revised exposure estimation, the mRPI was reduced to 0.9–3.1 (mean LB-95P UB). Since the value is above 1, potential health risks on the development associated with this mixture cannot be excluded. The individual RPQs of each FA with the original exposure estimate, but also with the refined exposure estimate, are shown in Figure 4.

A more detailed description of the endpoints is given in the Supplementary Material Tables S1, S9 and S10.

## 4. Discussion

Health risks for only three endpoints (kidney, reproduction and developmental toxicity) were identified. Steviol glycosides (E 960 a–d) and glutamic acid/glutamates (E 620–625) were identified as the main risk drivers. The applied mRPI approach that was originally developed to assess the mixture of food contaminants is also applicable for the mixture assessment of FAs [39].

### 4.1. Exposure Assessment

To estimate the dietary uptake of FAs, the EFSA usually uses the brand-loyal and general population approach. For the brand-loyal population, it is assumed that the individual is a long-term brand-loyal consumer of one food category containing the FA at the highest reported percentile and is non-brand loyal to the other food categories in the diet, which contain the FA at a mean concentration level [40]. In this assessment, the exposure was estimated on a food-item level for the general population. The exposure of brand-loyal users only was not assessed. In Table 7, our dietary exposure estimates are compared to those reported by the EFSA, with the latter being slightly higher overall. Differences between the two exposure assessments can be attributed to the use of different consumption and occurrence data, as the EFSA’s estimates reflect exposure at the European level (e.g., E 100, E 104, E 122, E 124, E 129, E 155, E 160 e, E 200–202, E 951, E 960 a–d), whereas the present study focused on the Austrian population. Additionally, not all analytical occurrence data were relevant and were not used for this assessment, e.g., samples of food colors or their salts.

Since estimated exposures to glutamate and steviol were exceptionally high, we re-examined the occurrence data and identified samples with very high concentrations that appeared implausible for the intended use. For glutamate, 2163 samples were excluded in the refined assessment, since their high concentrations were considered more likely to reflect natural occurrence of the amino acid glutamic acid than its use as a FA. For steviol, four tea samples with exceptionally high concentrations were suspected to represent measurements of tea leaves rather than tea extracts and were therefore excluded.

After the exclusion of those samples, the risk was noticeably reduced. Since most of the data were left censored, the results of the lower bound mean estimation seem to be the most realistic.

### 4.2. Mixture Assessment

After the refinement of the exposure assessment, only for the kidney endpoint (mRPI: 0.3–1.6) and developmental toxicity endpoint (mRPI: 0.9–3.1) was a potential health concern identified. For the other endpoints, no risk was identified.

In general, selection of the FAs for this mixture assessment was based on previously evaluated exposure estimations by the EFSA, prioritizing substances with exposures constituting substantial proportions of the ADI. Furthermore, FAs with high levels in certain food categories, which are not considered relevant for Austrian food consumption habits, were not prioritized. Since our analyses are not based on duplicate diet studies, the evaluated mixtures are not real-life mixtures but rely on several assumptions such as the use of mean concentrations of specific substances in specific food categories.

Furthermore, consumption behavior, but also brand loyalty, have an impact on the individual FA exposure, which can lead to an infinite number of FA mixture possibilities. However, we can only conclude on the likelihood that humans are exposed to this evaluated mixture. Hence, we used the Mintel database, which shows that for some FAs, it is more likely that they occur in the same product and are therefore more likely to be consumed together throughout the day than others. For the kidney endpoint, we identified that two or three of the corresponding FAs (E 200–202, E 220–229, E 249–250, E 338–343; E 450–452, E 960 a–d, E 968) were present together in several products. At least two of the selected FAs (E 960 a–d, E 220–229, E 620–625 and E 210–213) for the developmental toxicity endpoint occurred together in the same food items. This database and the available analytical data indicate that substances like brown HT (E 155), azorubine (E 122) or ponceau (E 124) are not that frequently used and may not be as relevant for this mixture assessment as the other considered substances. In a French cohort study, it was shown that in the 50 most frequently consumed FAs, some of our selected FAs were also given (such as phosphates, curcumin, aspartame, annatto extracts, nitrite, glutamate, carrageen), and within the 90 most frequently consumed FAs, sorbates, sulphates, steviol glycosides, sucrose of the fatty acids, ß-apo-8′ carotenal, quinoline yellow, Allura Red AC, and indigo carmine were also reported [41]. The findings of this study, supported by the analysis of the Mintel database, indicate that the chosen FAs are regularly consumed together to a significant extent through the diet. This supports the conclusion that some of the identified FAs may have an impact on mixture toxicity.

For the safety evaluation of a FA, endpoints like body weight change, clinical chemistry, gross and histopathological organ toxicity, carcinogenicity and reproduction/developmental toxicity will be assessed through genotoxicity studies, a subchronic 90-day or longer animal study and, in some cases, an extended generational study [42]. This study follows those principles. However, it is difficult to compare our results with previously published literature, since most of the FA mixtures are mainly assessed through one epidemiological study (NutriNet-Santé Cohort), which has its focus on diseases such as type 2 diabetes, cancer or cardiovascular diseases. Here, two FA mixtures were identified containing selected emulsifiers, preservatives, food colors and non-nutritive sweeteners that correlate with type 2 diabetes. A mechanistic plausibility could be that, e.g., some emulsifiers interact with mucus-microbiota and cause disruption at the gut barrier and that specific non-nutritive sweeteners cause microbiome-dependent glycemic effects [4]. Recent research also investigated the impact of FAs, in particular emulsifiers and sweeteners, on the microbiome [4,8,43,44,45]. From a regulatory perspective, it is difficult to assess the impact on the microbiome, since this research is in an early stage and therefore cannot be used as a basis to draw a robust regulatory conclusion on the impact on the microbiome. While this is an endpoint that should be considered for the assessment of FAs on an individual but also on a mixture level, further research is needed. Based on the outcome of the NutriNet-Santé study, an in vitro cytotoxicity and genotoxicity study was conducted with the identified six mixtures and their corresponding intake. A total of 32 FAs were tested on an individual level but also in the presence of the identified six different mixtures at five concentrations over a 24 h treatment. Two tested mixtures were genotoxic at the highest dose tested, while one mixture was cytotoxic. The cytotoxic effects were also observed in individual testing for the substances that were tested in this mixture. Nevertheless, in one mixture that contained high concentrations of modified starch (E 1442), citric acid (E 330), sodium carbonates (E 500) and monosodium glutamate (E 621), a synergistic effect was observed [46]. There are many more publications available for ultra-processed food, where FAs are widely and highly used, indicating similar health concerns [43,47]. These studies have not been considered, since other nutritional factors such as excess energy density, fiber and micronutrient displacement can also have health impacts, and this study focuses solely on the impact of FAs.

Overall, it can be concluded that there is some mechanistic evidence that certain FA mixtures can have a negative impact on human health by causing some low-grade microbiome-mediated metabolic effects through pathways such as gut microbiota composition, mucus barrier integrity or causing low-grade inflammation, which lead to metabolic diseases that were not systematically evaluated in the toxicological studies, which are used for regulatory risk assessment [4]. However, this does not imply that the current set of regulatory ADI’s are not safe. It rather underlines that some mixture effects might not be fully captured in a single substance risk assessment and need to be further researched.

### 4.3. Uncertainty and Limitations

This study only applies to the Austrian adult population. The exposure was estimated using a deterministic approach, hence on the assumption that specific products contain mean concentrations of specific substances. One possibility to reduce associated uncertainties would be the application of a probabilistic exposure assessment approach. However, since only two FAs were identified as risk drivers, collecting more data on occurrence appears more significant than a refinement of the exposure assessment.

Currently, the food consumption database lacks specific information, e.g., whether a consumed dish was made from scratch or with ready-to-use ingredients. In the present study, it was assumed that, for example, in every consumed soup, a stock cube was used, which potentially leads to overestimations in the exposure assessment. Another uncertainty is that we received the data coded in FoodEx2. For certain samples, we identified problems combining them with our food consumption data. This also might cause a slight over- or underestimation of the exposure.

Furthermore, some FAs had to be excluded from this assessment due to the lack of analytical data. However, some of these FAs might be of relevance, e.g., carrageenan, which is quite frequently used and might be a considerable contributor to mixture toxicity. This potentially leads to an underestimation of the real mixture risk. Nevertheless, this outcome helps prioritize certain FAs for intensified monitoring, where analytical data are missing or incomplete, in order to close data gaps for future mixture assessments.

Another limitation of this study is that based on publicly available toxicity data, it is only possible to identify common effects at the organ level. The current accessible data situation does not allow identification of common adverse outcome pathways (AOPs) or modes of action (MOA), as it is suggested as a refinement by the EFSA [48]. Other accessible tools, such as the chemical mixture calculator developed by the DTU, are limited to food colors and therefore may be excluded from further refinement of the present assessment [49].

In addition, consideration of toxicological interactions between certain FAs that may exacerbate or attenuate negative health impacts or diseases, hence synergistic or antagonistic effects, is currently not possible due to lack of data.

The default assumption of concentration addition was applied to assess combined effects of FAs within the mixtures.

## 5. Conclusions

The aim of this study was to assess if the available data allow us to draw conclusions about a potential mixture risk associated with the consumption of FAs. The current data availability in Austria did not allow the identification of a mixture that is consumed regularly. This was primarily due to limitations in the food consumption data, which did not provide sufficient information to determine which specific food products and their brands were consumed regularly. Moreover, not all FAs are analytically measured in food products. Therefore, we were not able to identify a mixture according to its actual occurrence. To address this limitation, a novel approach was tested. FAs were prioritized according to their ADI contributions and their use in specific food categories. The prioritized FAs were then grouped by toxicological endpoints. Combinations of specific food colors, preservatives, flavor enhancers and sweeteners were identified, which might be of relevance regarding mixture effects on common target organs. For some of these FA combinations, the actual occurrences in a mixture were partly confirmed by databases and literature. Potential health concerns were identified for nephrotoxicity and developmental toxicity. A refinement of these endpoints was not possible due to limitations of the current toxicological data. Available peer-reviewed literature indicates that combinatory effects of FAs through different mechanisms may regularly contribute to the development of diseases such as type 2 diabetes. In a single-substance evaluation, those mechanisms cannot be displayed; therefore, further research is needed to draw conclusions about the mixture effects of FAs. However, the study of mixture effects requires the identification of real-life food additive mixtures. The forthcoming EU-wide monitoring program could contribute to addressing this need by collecting data on the occurrence of FAs at the product level. Despite the limitations mentioned, the applied novel approach represents a promising screening strategy for identifying and prioritizing FA mixtures of potential health concern. Further development and validation of the methodology, by using improved occurrence data from real-life food products, would be highly desirable to enable a more realistic view of the risks of FA mixtures.

## Figures and Tables

**Table 1:** Selected food additives.

Curcumin (E 100)	Potassium/sodium nitrates (E 251–252)
Tartrazine (E 102)	Erythorbic acid and sodium erythorbate (E 315–316)
Quinoline Yellow (E 104)	Tartaric acid/tartrates (E 334–337; 354)
Cochineal, carminic acid, carmines (E 120)	Phosphates (E 338–343; E 450–452)
Azorubine (E 122)	Carrageenan and processes Eucheuma Seaweed (E 407; E407 a)
Ponceau (E 124)	Polysorbate (E 432–436)
Allura Red AC (E 129)	ß-cyclodextrin (E 459)
Patent Blue V (E 131)	Diglycerides of fatty acids (E 472 d–f)
Indigo carmine (E 132)	Sucrose esters of fatty acids (E 473)
Brilliant Blue (E 133)	Sodium and calcium stearoyl-2-lactylates (E 481–482)
Caramel colors (E 150 (a,b,c,d))	Sorbitan esters (E 491–495)
Brown HT (E 155)	Hydrochloric acid and its potassium, calcium and magnesium salts (E 507–511)
Annatto extracts (E 160 b)	Glutamic acid/glutamates (E 620–625)
Lycopene (E 160 d)	Acesulfame K (E 950)
ß-apo-8′-carotenal (E 160 e)	Aspartame (E 951)
Lutein (E 161 b)	Cyclamates (E 952)
Sorbic acid and its potassium salt (E 200–202)	Saccharin (E 954)
Benzoic acid and sodium/potassium benzoate (E 210–213)	Sucralose (E 955)
Sulfur dioxide/sulfites (E 220–229)	Steviol glycosides (E 960 a–d)
Ethyl lauroyl arginate (E 243)	Erythritol (E 968)
Potassium/sodium nitrite (E 249–250)	Quillaia extract (E 999)

**Table 2:** Food additives and their respective toxicological endpoints.

Liver	Kidney	Gastro-Intestinal	Developmental	Reproduction	Hematology
Ponceau (E 124)	Ponceau (E 124)	Curcumin (E 100)	Curcumin (E 100)	Quinoline Yellow (E 104)	Azorubine (E 122)
Brown HT (E 155)	Brown HT (E 155)	Azorubine (E 122)	Quinoline Yellow (E 104)	Allura Red AC (E 129)	Ponceau (E 124)
Annatto extracts (E 160 b)	Annatto extracts (E 160 b)	Brown HT (E 155)	Ponceau (E 124)	Brown HT (E 155)	Indigo carmine (E 132)
ß-apo-8′-carotenal (E 160 e)	Sorbic acid and its potassium salt (E 200–202)	Phosphates (E 338–343; E 450–452)	Allura Red AC (E 129)	ß-cyclodextrin (E 459)	Brown HT (E 155)
Sorbic acid and its potassium salt (E 200–202)	Sulfur dioxide/sulfites (E 220–229)	Carrageenan and processes Eucheuma Seaweed (E 407; E407 a)	Lycopene (E 160 d)	Steviol glycosides (E 960 a–d)	Sulfur dioxide/sulfites (E 220–229)
Benzoic acid and sodium/potassium benzoate (E 210–213)	Potassium/sodium nitrite (E 249–250)	ß-cyclodextrin (E 459)	Sorbic acid and its potassium salt (E 200–202)		Ethyl lauroyl arginate (E 243)
Carrageenan and processes Eucheuma Seaweed (E 407; E407 a)	Phosphates (E 338–343; E 450–452)		Benzoic acid and sodium/potassium benzoate (E 210–213)		Potassium/sodium nitrite (E 249–250)
ß-cyclodextrin (E 459)	ß-cyclodextrin (E 459)		Ethyl lauroyl arginate (E 243)		Sorbitan esters (E 491–495)
Sorbitan esters (E 491–495)	Sorbitan esters (E 491–495)		Carrageenan and processes Eucheuma Seaweed (E 407; E407 a)		
	Aspartame (E 951)		ß-cyclodextrin (E 459)		
	Steviol glycosides (E 960 a–d)		Glutamic acid/glutamates (E 620–625)		
	Erythritol (E 968)		Aspartame (E 951)		
			Steviol glycosides (E 960 a–d)		

**Table 3:** Occurrence data from the analyzed food additives in µg/g.

Food Additive	Data Origin	*n*	*n* < LOQ	Mean	Std	Median	95 P	Min–Max
Curcumin (E 100)	EFSA	1108	103	1537.4	18,935	0.00	30.9	0.0–399,000
Quinoline Yellow (E 104)	AT	1053	24	32.3	806.9	0.00	0.00	0.00–25,600
Azorubine (E 122)	AT	1054	35	171.8	2386	0.00	0.00	0.00–52,103
Ponceau (E 124)	AT	1130	60	519.8	14,516.5	0.00	7.4	0.00–487,016
Allura Red AC (E 129)	AT	1054	53	19.9	346.4	0.00	0.9	0.00–8283
Indigo carmine (E 132)	AT	1052	18	6.8	169.6	0.00	0.00	0.00–5396
Brown HT (E 155)	EFSA	1075	8	1.5	47	0.00	0.00	0.00–1540
Annatto extracts (E 160 b)	EFSA	96	17	1.3	3.4	0.00	9.8	0.00–15.1
ß-apo-8′-carotenal (E 160 e)	EFSA	78	0	0.00	0.00	0.00	0.00	0.00
Sorbic acid and its potassium salt (E 200–202)	AT	3307	868	177.6	544.7	0.00	1525.9	0.00–14,503.3
Benzoic acid and sodium/potassium benzoate (E 210–213)	AT	3305	382	48.7	221.1	0.00	235.9	0.00–3802.5
Sulfur dioxide/sulfites (E 220–229)	AT	1113	245	140.2	4222.7	0.00	55.75	0.00–140,864.1
Potassium/sodium nitrite (E 249–250)	AT	3095	1505	9.98	15.3	0.00	40.9	0.00–189.15
Phosphates (E 338–343; E 450–452)	EFSA	2526	201	167.8	1026.1	0.00	524.5	0.00–20,500
Glutamic acid/glutamates (E 620–625)	EFSA	37,279	33,227	5433.9	53,591.1	480	12,977.9	0.00–6,279,500
Refined Glutamic acid/glutamates (E 620–625)	EFSA	35,116	31,378	4015.8	52,655.1	490	10,035.25	0.00–6,279,500
Aspartame (E 951)	AT	2070	122	324	8786.3	0.00	28.5	0.00–2,822,000
Steviol glycosides (E 960 a–d)	EFSA	3391	1285	2045.6	15,889.8	0.00	2080	0.00–269,000
Refined Steviol glycosides (E 960 a–d)	EFSA	3387	1281	1991.2	15,792	0.00	1978.5	0.00–269,000
Erythritol (E 968)	AT	366	34	53,209	215,297	0.00	593,362.5	0.00–1,032,240

AT: Austrian Data, EFSA: Data was provided by EFSA incl. Austrian data, *n*: number of samples, *n* < LOQ: number of samples below the Limit of Quantification, std: standard deviation, 95P.: 95th Percentile.

**Table 4:** The estimation of the food additives uptake of Austrian adults in mg/kg bw/d.

Food Additive		Mean	Std	Median	95P.	Min–Max
Curcumin (E 100)	LB	0.382	2.044	0.007	1.909	0.0–64.16
	MB	0.398	2.044	0.029	1.914	0.0–64.17
	UB	0.415	2.044	0.050	1.926	0.0–64.19
Quinoline Yellow (E 104)	LB	0.001	0.001	0.000	0.003	0.0–0.02
	MB	0.008	0.007	0.006	0.023	0.0–0.05
	UB	0.016	0.014	0.012	0.046	0.0–0.11
Azorubine (E 122)	LB	0.004	0.008	0.000	0.019	0.0–0.07
	MB	0.012	0.012	0.008	0.036	0.0–0.10
	UB	0.019	0.018	0.014	0.056	0.0–0.13
Ponceau (E 124)	LB	0.006	0.012	0.000	0.030	0.0–0.10
	MB	0.014	0.016	0.008	0.046	0.0–0.12
	UB	0.021	0.021	0.015	0.064	0.0–0.15
Allura Red AC (E 129)	LB	0.040	0.092	0.007	0.200	0–1.71
	MB	0.047	0.093	0.014	0.206	0–1.72
	UB	0.053	0.094	0.022	0.209	0–1.73
Indigo carmine (E 132)	LB	0.000	0.000	0.000	0.003	0.0–0.01
	MB	0.008	0.007	0.006	0.022	0.0–0.05
	UB	0.016	0.014	0.012	0.044	0.0–0.11
Brown HT (E 155)	LB	0.001	0.011	0.000	0.000	0.0–0.62
	MB	0.004	0.011	0.003	0.008	0.0–0.63
	UB	0.008	0.012	0.007	0.015	0.0–0.65
Annatto extracts (E 160 b)	LB	0.001	0.002	0.000	0.004	0.0–0.01
	MB	0.001	0.002	0.001	0.005	0.0–0.01
	UB	0.002	0.002	0.001	0.005	0.0–0.01
ß-apo-8′-carotenal (E 160 e)	LB	0.000	0.000	0.000	0.000	0.0–0.0
	MB	0.001	0.002	0.000	0.004	0.0–0.02
	UB	0.002	0.003	0.001	0.009	0.0–0.04
Sorbic acid and its potassium salt (E 200–202)	LB	0.411	0.420	0.298	1.204	0.0–4.36
	MB	0.433	0.422	0.319	1.223	0.0–4.36
	UB	0.456	0.425	0.341	1.259	0.0–4.36
Benzoic acid and sodium/potassium benzoate (E 210–213)	LB	0.097	0.200	0.026	0.459	0.0–3.20
	MB	0.123	0.202	0.055	0.481	0.0–3.23
	UB	0.149	0.205	0.084	0.519	0.0–3.27
Sulfur dioxide/sulfites (E 220–229)	LB	0.007	0.023	0.002	0.028	0.0–0.46
	MB	0.009	0.023	0.004	0.030	0.0–0.47
	UB	0.011	0.024	0.005	0.035	0.0–0.47
Potassium/sodium nitrite (E 249–250) *	LB	0.002	0.004	0.000	0.010	0.0–0.05
	MB	0.003	0.004	0.001	0.011	0.0–0.05
	UB	0.003	0.005	0.001	0.013	0.0–0.05
Phosphates (E 338–343; E 450–452)	LB	1.574	1.987	0.778	5.68	0.0–14.15
	MB	2.028	2.030	1.328	6.03	0.0–14.15
	UB	2.482	2.130	1.848	6.64	0.0–15.10
Glutamic acid/glutamates (E 620–625)	LB	373.02	499.88	173.56	1434.87	0.99–3558.2
	MB	373.39	499.91	173.86	1435.43	1.04–3558.4
	UB	373.76	499.95	174.80	1436.00	1.06–3558.7
Aspartame (E 951)	LB	0.049	0.132	0.000	0.249	0.0–1.92
	MB	0.118	0.150	0.078	0.343	0.0–1.95
	UB	0.187	0.180	0.142	0.496	0.0–2.04
Steviol glycosides ** (E 960 a–d)	LB	6.138	14.629	0.011	37.609	0.0–148.81
	MB	6.148	14.627	0.012	37.612	0.0–148.82
	UB	6.158	14.626	0.013	37.616	0.0–148.82
Erythritol (E 968)	LB	6.951	13.881	0.131	35.435	0.0–140.80
	MB	8.183	13.953	2.236	37.377	0.0–141.56
	UB	9.415	14.075	3.969	38.026	0.0–142.33

LB: Lower bound, MB: Medium Bound, UB: Upper Bound, std: standard deviation, 95P: 95th percentile, * expressed as nitrite ions, ** expressed as steviol glycoside equivalents (conversion factor of 0.4).

**Table 5:** Number of products and their corresponding food categories that were on the Austrian market between the years 2015 and 2025 according to the Mintel database.

Food Additive	*n*	Food Categories
Curcumin (E 100)	320	Confectioneries (e.g., pastilles, gum, chocolate desserts, dairy ice cream, and frozen yogurt), baking mixes and ready-to-eat meals
Quinoline Yellow (E 104)	11	Beverages (non-alcoholic and alcoholic aperitifs, liqueur, cider) and protein bars
Azorubine (E 122)	8	Beverages (e.g., soft drinks and non-alcoholic aperitif), liqueur and sweet snacks
Ponceau (E 124)	5	Beverages (e.g., non-alcoholic aperitif) and alcoholic beverages (e.g., aperitif and liqueur) and sweet snacks
Allura Red AC (E 129)	13	Beverages (e.g., energy drinks, soft drinks and non-alcoholic aperitif) and liqueur
Indigo carmine (E 132)	17	Confectioneries (chocolate, gummy candy) and cake decorations
Brown HT (E 155)	1	Cake decorations
Annatto extracts (E 160 b)	40	Cheese, chips, cereals, seasonal products (e.g., Easter eggs) and desserts (cake and ice cream)
ß-apo-8′-carotenal (E 160 e)	22	Confectioneries (chocolate, jelly gums, cake) and beverages (soft drinks and energy drinks)
Sorbic acid and its potassium salt (E 200–202)	1138	Confectioneries (chocolate, cakes, desserts, biscuits), beverages (soft drinks, syrup, energy drinks), ready-to-eat meals (salad, meat preparations, soups) sauces, bread, cream cheese and potato products
Benzoic acid and sodium/potassium benzoate (E 210–213)	253	Beverages (syrup, non-alcoholic drinks, soft drinks, energy drinks), sauces and ready-to-eat meal (salads, meat dishes)
Sulfur dioxide/sulfites (E 220–229)	465	Ready-to-eat meals, shrimp/prawns, sauces and dressings, dried fruits and savory snacks (nuts, crackers)
Potassium/sodium nitrite (E 249–250)	1857	Meat products or when meat was used in ready-to-eat meals
Phosphates (E 338–343; E 450–452)	2105	Meat products, shrimps/prawns, confectionary (e.g., biscuits, cakes, ice cream, chocolate) ready-to-eat meals, savory snacks, plant-based products (drinks, yogurt) and milk drinks
Glutamic acid/glutamates (E 620–625)	505	Ready-to-eat meals, seasoning, savory snacks, meat products, soup stocks and sauces
Aspartame (E 951)	127	Beverages (e.g., soft drinks, juices, milk drinks) and confectioneries (e.g., chewing gum)
Steviol glycosides (E 960 a–d)	146	Confectioneries (e.g., hard candies, chewing gum, ice cream), beverages (e.g., soft drinks, syrup, milk drinks, protein powders) sauces and snacks (protein bars)
Erythritol (E 968)	100	Beverages (e.g., soft drinks, dairy drinks), confectioneries (e.g., chocolate bars, ice cream, jelly gums), sauces and dairy products (e.g., yogurt)

**Table 6:** Revised exposure estimation for glutamates and steviol glycosides in mg/kg bw/d.

Food Additive		Mean	Std	Median	95P.	Min–Max
Glutamic acid/glutamates (E 620–625)	LB	13.762	18.484	10.461	32.384	0.00–482.24
	MB	13.946	18.503	10.671	32.611	0.00–482.38
	UB	14.129	18.523	10.845	32.825	0.00–482.52
Steviol glycosides * (E 960 a–d)	LB	0.506	0.988	0.113	2.586	0.00–10.14
	MB	0.516	0.988	0.123	2.589	0.00–10.15
	UB	0.526	0.988	0.132	2.595	0.00–10.15

LB: Lower bound, MB: Medium Bound, UB: Upper Bound, std: standard deviation, 95P: 95th percentile, * expressed as steviol equivalents.

**Table 7:** Comparison of the estimated dietary exposure (LB mean) with the estimation for the general non-brand-loyal population published by EFSA. Both estimates are given in mg/kg bw/d.

Food Additive	Exposure Estimation (LB-Mean)	EFSA Exposure Estimation General Population (Mean)
Curcumin (E 100)	0.382	0.2
Quinoline Yellow (E 104)	0.001	0.01
Azorubine (E 122)	0.004	0.2
Ponceau (E 124)	0.006	0.05
Allura Red AC (E 129)	0.040	0.2
Indigo carmine (E 132)	0.000	0.02
Brown HT (E 155)	0.001	0.03
Annatto extracts (E 160 b)	0.001	0.02
ß-apo-8′-carotenal (E 160 e)	0.000	0.1
Sorbic acid and its potassium salt (E 200–202)	0.411	0.8
Benzoic acid and sodium/potassium benzoate (E 210–213)	0.097	1.6
Sulfur dioxide/sulfites (E 220–229)	0.007	0.31
Potassium/sodium nitrite (E 249–250) *	0.002	0.02
Phosphates (E 338–343; E 450–452)	1.574	3.7
Glutamic acid/glutamates (E 620–625)	373.02	19
Revised Glutamic acid/glutamates (E 620–625)	13.762	19
Aspartame (E 951)	0.049	8.5
Steviol glycosides ** (E 960 a–d)	6.138	1.1
revised Steviol glycosides ** (E 960 a–d)	0.506	1.1
Erythritol (E 968)	6.951	170

* expressed as nitrite ions, ** expressed as steviol equivalents.

## Data Availability

The data presented in this study is partly publicly available; the food additive occurrence data can be accessed by a public data request at the EFSA.

## Supplementary Materials

The following Supporting Information can be downloaded at https://www.mdpi.com/article/10.3390/toxics14100929/s1, Table S1. Detailed description of the toxicological effects per endpoint and used food additives; Table S2. Detailed overview of Reference Point Quotient of the food additives and its modified Reference Point Index (mRPI) for the sensitive endpoint liver; Table S3. Detailed overview of Reference Point Quotient of the food additives and its modified Reference Point Index (mRPI) for the sensitive endpoint gastrointestinal toxicity; Table S4. Detailed overview of Reference Point Quotient of the food additives and its modified Reference Point Index (mRPI) for the sensitive endpoint hematotoxicity; Table S5 Detailed overview of Reference Point Quotient of the food additives and its modified Reference Point Index (mRPI) for the sensitive endpoint kidney; Table S6. Detailed overview of Reference Point Quotient of the food additives and its modified Reference Point Index (mRPI) for the sensitive endpoint kidney with revised exposure data; Table S7. Detailed overview of Reference Point Quotient of the food additives and its modified Reference Point Index (mRPI) for the sensitive endpoint reproduction toxicity; Table S8. Detailed overview of Reference Point Quotient of the food additives and its modified Reference Point Index (mRPI) for the sensitive endpoint reproduction toxicity with the revised exposure data; Table S9. Detailed overview of Reference Point Quotient of the food additives and its modified Reference Point Index (mRPI) for the sensitive endpoint development toxicity; Table S10. Detailed overview of Reference Point Quotient of the food additives and its modified Reference Point Index (mRPI) for the sensitive endpoint development toxicity revised exposure data.

## Abbreviations

The following abbreviations are used in this manuscript:

ADI	Acceptable Daily Intake
EFSA	European Food Safety Authority
FA	Food Additive
JECFA	Joint FAO/WHO Expert Committee on Food Additives
mRPI	modified Reference Point Index
RPQ	Reference Point Quotient
