# Supplementary material for: Identification of a Potential Food Additive Mixture and Health Risk Assessment Using the Modified Reference Point Index (mRPI)
Source: Toxics. 2026 Oct 20;14(10):929. doi: 10.3390/toxics14100929 (PMC13709900; doi:10.3390/toxics14100929)
Supplement: Supplementary file 1 [file toxics-14-00929-s001.zip › toxics-4571949-supplementary.pdf]

Table S1. Detailed description of the toxicological effects per endpoint and used food additives

Endpoint	Food additive	Effects on the Endpoint	Reference Point and Uncertainty Factor (UF)
Liver	Ponceau (E 124)	increased incidence of foamy reticulo-endothelial cells in the liver [14]	NOAEL: 375 mg/kg bw/d<br>UF: 100
	Brown HT (E 155)	increased incidence of leucocyte infiltration in the liver [15,16]	NOAEL: 143 mg/kg bw/d<br>UF:100
	Annatto extracts (E 160 b)	increased absolute and relative liver weight, hepatocellular hypertrophy [17,18]	NOAEL: 63 mg/kg bw/d<br>UF: 200 (due to subchronic study)
	ß-apo-8'-carotenal (E 160 e)	increased liver weight, serum ALT and AST, inflammatory cell foci in the liver [19,20]	NOAEL: 30 mg/kg bw/d<br>UF: 100
	Sorbic acid and its potassium salt (E 200-202)	relative liver weights increased [21]	NOAEL: 630 mg/kg bw/d<br>UF: 100
	Benzoic acid and sodium/potassium benzoate (E 210-213)	increased relative liver weight on post-mortem examination [22,23]	NOAEL: 160 mg/kg bw/d<br>UF: 100<br>Lower than ADI, therefore ADI was chosen (5 mg/kg bw/d)
Kidney	Ponceau (E 124)	increased incidence glomerulonephrosis [14]	NOAEL: 70 mg/kg bw/d<br>UF: 100
	Brown HT (E 155)	increased kidney weight [15,16]	NOAEL: 250 mg/kg bw/d<br>UF:100
	Annatto extracts (E 160 b)	changes in renal function: slightly increased kidney weights, increased plasma creatinine and urea concentrations, slightly reduced urinary output and slightly low pH values [17,18]	NOAEL: 30<br>UF 200 due to subchronic studies. Lower than ADI, therefore ADI was chosen (0.3 mg/kg bw/d)
	ß-apo-8'-carotenal (E 160 e)	eosinophilic droplet formation in outer renal cortices, increase in creatinine levels [19,20]	LOAEL: 10 mg/kg bw/d<br>UF: 200 (decided by the EFSA Panel), it's the ADI of 0.05 mg/kg bw/d
	Sorbic acid and its potassium salt (E 200-202)	increased relative kidney weights [21]	NOAEL: 630 mg/kg bw/d<br>UF: 100
	Sulfur dioxide/ sulfites (E 220-229)	reduced albumin and drop blood glucose levels [30]	LOAEL: 9.4 mg/kg bw/d<br>UF: 2000 (due to subchronic study and LOAEL to NOAEL), Lower than ADI, therefore ADI was chosen (0.7 mg/kg bw/d)

	Potassium / sodium nitrite (E 249-250)	decreased plasma aldosterone and corticosterone [31]	NOAEL: 7.3 mg/kg bw/d<br>UF: 200 (due to subchronic study), Lower than ADI, therefore ADI was chosen (0.07 mg nitrite ion/kg bw/d)
	Phosphates (E 338-343; E 450-452)	inflammatory changes in the kidney [27,28]	NOAEL: 45 mg/kg bw/d<br>UF: 200 (due to subchronic study), Lower than ADI, therefore ADI was chosen (42 mg/kg bw/d)
	Aspartame (E 951)	renal changes in males (renal pigment deposit in tubular and pelvic epithelial cells, focal hyperplasia of renal pelvic epithelim, tubular degeneration) [32]	NOAEL: 4000<br>UF 100
	Steviol glycosides (E 960 a-d)	microscopical changes in kidneys, dilation of proximal and distal convoluted tubules, hyaline content in tubules, desquamation of the epithelial cells of some proximal convoluted tubules [33]	NOAEL: 383 mg steviol equivalents/ kg bw/d<br>UF: 100<br>It's the ADI
	Erythritol (E 968)	increased water consumption, increased plasma alkaline phosphatase activity, diuresis, and increased urinary calcium excretion [34]	NOEL:1400 mg/kg bw/d<br>UF: 100<br>Its lower than ADI, therefore ADI was chosen (500 mg/kg bw/d)
GIT	Curcumin (E 100)	Gastrointestinal irritation (ulcers, hyperplasia and inflammation) [24,25]	NOAEL: 440 mg/kg bw/d<br>UF:100
	Azorubine (E 122)	alter body weights and caecum weights<br>[26]	NOAEL:400mg/kg bw/d<br>UF:100<br>Its the ADI
	Brown HT (E 155)	caecum enlargement, brown coloration of the lymph nodes and some areas of gastro-intestinal tract [15,16]	NOAEL: 250 mg/kg bw/d<br>UF:100
	Phosphates (E 338-343; E 450-452)	hypertrophy and haemorrhages of cardiac/pyolirc border of the stomach in male and females [27,28]	NOAEL: 300 mg/kg bw/d<br>UF:200 due to subchronic study. Lower than ADI, therefore ADI was chosen (42 mg/kg bw/d)
Developm ental tox	Curcumin (E 100)	decreased body-weight gain in the F2 generation<br>[24,25]	NOAEL: 250 mg/kg bw/d<br>UF: 100<br>RP was used to derived the ADI of 3 mg/kg bw/d, therefore the ADI was used
	Quinoline Yellow (E 104)	decreased body weight gain of F1 pups and reduced pup viability [35,36]	NOAEL: 50 mg/kg bw/d<br>UF: 100
	Ponceau (E 124)	decreased weight gain, increased caecum weight, high concentration of proteins in the urine (female), increased kidney and testes weight (males) [14]	NOAEL: 500 mg/kg bw/d<br>UF:100

	Allura Red AC (E 129)	Increased occurrence of reduced ossification of the hyoid in fetuses [37]	NOAEL: 546 mg/kg bw/d<br>UF: 100<br>Lower than ADI, therefore ADI was chosen (7 mg/kg bw/d).
	Sorbic acid and its potassium salt (E 200-202)	reduced body weight in F1 and F2 pups, effects on morphological landmarks and physical development in F1 pups, effect on anogenital distance [21]	NOAEL: 300 mg/kg bw/d<br>UF: 100
	Benzoic acid and sodium/potassium benzoate (E 210-213)	reduced fetal weight, malformations, fetal developmental variations, variations due to retarded development [22,23]	NOAEL: 160 mg/kg bw/d<br>UF: 100<br>Lower than ADI, therefore ADI was chosen (5 mg/kg bw/d)
	Glutamic acid/glutamates (E 620-625)	delayed early swimming development, diminished rearing frequency in the open field, altered active avoidance acquisition and extinction, and prolonged day-2 passive avoidance retention were observed, trend towards decreased running wheel activity [38]	NOAEL 3200 mg/kg bw/d<br>UF:100<br>Represents the group ADI of 30 mg/kg bw/d
	Aspartame (E 951)	Significant decrease of the number of viable pups per litter at birth and pup survival until weaning, significant body weight suppression of the pups at birth [32]	NOAEL: 2000 mg/kg bw/d<br>UF:100<br><br>Lower than ADI, therefore ADI was chosen (40 mg/kg bw/d)
	Steviol glycosides (E 960 a-d)	statistically significantly increased mortality rate and statistically significantly decreased fetal weight in the three highest dose levels and statistically significantly lower number of foetuses per litter [33]	NOAEL: 383 mg steviol equivalents/ kg bw/d<br>UF: 100<br>It's the ADI
Reproduction tox	Quinoline Yellow (E 104)	increased uterus weight [35,36]	NOAEL: 250 mg/kg bw/d<br>UF: 100
	Allura Red AC (E 129)	decreased reproductive success [37]	LOAEL: 1250 mg/kg bw/d<br>UF: 1000 (due to LOAEL- NOAEL extrapolation)<br>Lower than ADI, therefore ADI was chosen (7 mg/kg bw/d)
	Brown HT (E 155)	increase in cystic ovaries [15,16]	NOAEL: 143 mg/kg bw/d<br>UF: 100
	Steviol glycosides (E 960 a-d)	decreased absolute left ovary weights [33]	NOAEL: 383 mg steviol equivalents/ kg bw/d<br>UF: 100<br>It's the ADI
Haematology	Azorubine (E 122)	decrease of haemoglobin levels; decreased packed cell volume [26]	NOAEL: 375 mg/kg bw/d<br>UF: 100<br>Its the ADI
	Ponceau (E 124)	decreased erythrocytes [14]	NOAEL: 300 mg/kg bw/d<br>UF:200 (due to subchronic study)

	Indigo carmine (E 132)	slight anaemia [29]	NOAEL: 600 mg/kg bw/d<br>UF: 100
	Brown HT (E 155)	decreased packed cell volume and total leucocyte count values [15,16]	NOAEL: 143 mg/kg bw/d<br>UF:100<br>Its the ADI
	Sulfur dioxide/sulfites (E 220-229)	reduced white blood cell count, reduced platelet count [30]	LOAEL: 9.4 mg/kg bw/d<br>UF: 2000 (due to subchronic and LOAEL to NOAEL extrapolation)<br>Lower than ADI, therefore ADI was chosen (0.7 mg/kg bw /d).
	Potassium / sodium nitrite (E 249-250)	increased Methaemoglobin [31]	ADI of 0.07 mg/kg bw/d was used

Table S2. Detailed overview of Reference Point Quotient of the food additives and its modified Reference Point Index (mRPI) for the sensitive endpoint liver

Food additive	Adults LB Mean	Adults LB 95P	Adults MB Mean	Adults MB 95P	Adults UB Mean	Adults UB 95P
Ponceau (E 124)	0.002	0.008	0.004	0.012	0.006	0.017
Brown HT (E 155)	0.001	0.000	0.003	0.005	0.006	0.011
Annatto extracts (E 160 b)	0.003	0.014	0.004	0.016	0.005	0.017
ß-apo-8'-carotenal (E 160 e)	0.000	0.000	0.004	0.015	0.007	0.030
Sorbic acid and its potassium salt (E 200-202)	0.065	0.191	0.069	0.194	0.072	0.200
Benzoic acid and sodium/potassium benzoate (E 210-213)	0.019	0.092	0.025	0.096	0.030	0.104
mRPI	0.09	0.30	0.11	0.34	0.13	0.38

Table S3. Detailed overview of Reference Point Quotient of the food additives and its modified Reference Point Index (mRPI) for the sensitive endpoint gastrointestinal toxicity

Food additive	Adults LB Mean	Adults LB 95P	Adults MB Mean	Adults MB 95P	Adults UB Mean	Adults UB 95P
Curcumin (E 100)	0.087	0.434	0.091	0.435	0.094	0.438
Azorubine (E 122)	0.001	0.005	0.003	0.009	0.005	0.014
Brown HT (E 155)	0.000	0.000	0.002	0.003	0.003	0.006
Phosphates (E 338-343; E 450-452)	0.037	0.135	0.048	0.144	0.059	0.158
mRPI	0.13	0.57	0.14	0.59	0.16	0.62

Table S4. Detailed overview of Reference Point Quotient of the food additives and its modified Reference Point Index (mRPI) for the sensitive endpoint hematotoxicity

Food additive	Adults LB Mean	Adults LB 95P	Adults MB Mean	Adults MB 95P	Adults UB Mean	Adults UB 95P
Azorubine (E 122)	0.001	0.005	0.003	0.010	0.005	0.015
Ponceau (E 124)	0.004	0.020	0.009	0.030	0.014	0.043
Indigo carmine (E 132)	0.000	0.000	0.001	0.004	0.003	0.007
Brown HT (E 155)	0.001	0.000	0.003	0.005	0.006	0.011
Sulfur dioxide/ sulfites (E 220-229)	0.010	0.039	0.013	0.042	0.015	0.048
Potassium / sodium nitrite (E 249-250)	0.031	0.149	0.038	0.168	0.045	0.189
mRPI	0.05	0.21	0.07	0.26	0.09	0.31

Table S5 Detailed overview of Reference Point Quotient of the food additives and its modified Reference Point Index (mRPI) for the sensitive endpoint kidney

Food additive	Adults LB Mean	Adults LB 95P	Adults MB Mean	Adults MB 95P	Adults UB Mean	Adults UB 95P
Ponceau (E 124)	0.009	0.043	0.020	0.065	0.031	0.092
Brown HT (E 155)	0.000	0.000	0.002	0.03	0.003	0.006
Annatto extracts (E 160 b)	0.003	0.015	0.005	0.016	0.006	0.018
ß-apo-8’-carotenal (E 160 e)	0.000	0.000	0.021	0.089	0.043	0.177
Sorbic acid and its potassium salt (E 200-202)	0.065	0.191	0.069	0.194	0.072	0.200
Sulfur dioxide/ sulfites (E 220-229)	0.010	0.040	0.013	0.043	0.015	0.050
Potassium / sodium nitrite (E 249-250)*	0.029	0.137	0.035	0.154	0.041	0.173
Phosphates (E 338-343; E 450-452)	0.037	0.135	0.048	0.144	0.059	0.158
Aspartame (E 951)	0.001	0.006	0.003	0.009	0.005	0.012

Steviol glycosides (E 960 a-d)*	1.603	9.820	1.605	9.820	1.608	9.821
Erythritol (E 968)	0.014	0.071	0.016	0.075	0.019	0.076
mRPI	**1.8**	**10.5**	**1.8**	**10.6**	**1.9**	**10.8**

*expressed as nitrite ions, **expressed as steviol equivalents

Table S6. Detailed overview of Reference Point Quotient of the food additives and its modified Reference Point Index (mRPI) for the sensitive endpoint kidney with revised exposure data

Food additive	Adults LB Mean	Adults LB 95P	Adults MB Mean	Adults MB 95P	Adults UB Mean	Adults UB 95P
Ponceau (E 124)	0.009	0.043	0.020	0.065	0.031	0.092
Brown HT (E 155)	0.000	0.000	0.002	0.003	0.003	0.006
Annatto extracts (E 160 b)	0.003	0.015	0.005	0.016	0.006	0.018
ß-apo-8'-carotenal (E 160 e)	0.000	0.000	0.021	0.089	0.043	0.177
Sorbic acid and its potassium salt (E 200-202)	0.065	0.191	0.069	0.194	0.072	0.200
Sulfur dioxide/ sulfites (E 220-229)	0.010	0.040	0.013	0.043	0.015	0.050
Potassium / sodium nitrite (E 249-250)*	0.029	0.137	0.035	0.154	0.041	0.173
Phosphates (E 338-343; E 450-452)	0.037	0.135	0.048	0.144	0.059	0.158
Aspartame (E 951)	0.001	0.006	0.003	0.009	0.005	0.012
Steviol glycosides (E 960 a-d)**	0.132	0.675	0.135	0.676	0.137	0.677
Erythritol (E 968)	0.014	0.071	0.016	0.075	0.019	0.076
mRPI	0.3	**1.3**	0.4	**1.5**	0.4	**1.6**

*expressed as nitrite ions, **expressed as steviol equivalents

Table S7. Detailed overview of Reference Point Quotient of the food additives and its modified Reference Point Index (mRPI) for the sensitive endpoint reproduction toxicity

Food additive	Adults LB Mean	Adults LB 95P	Adults MB Mean	Adults MB 95P	Adults UB Mean	Adults UB 95P
Quinoline Yellow (E 104)	0.000	0.001	0.003	0.009	0.006	0.018
Allura Red AC (E 129)	0.006	0.029	0.007	0.03	0.008	0.03
Brown HT (E 155)	0.001	0.000	0.003	0.005	0.006	0.011
Steviol glycosides (E 960 a-d)*	1.582	9.693	1.584	9.694	1.587	9.695
mRPI	**1.6**	**9.7**	**1.6**	**9.7**	**1.6**	**9.7**

*expressed as steviol equivalents

Table S8. Detailed overview of Reference Point Quotient of the food additives and its modified Reference Point Index (mRPI) for the sensitive endpoint reproduction toxicity with the revised exposure data

Food additive	Adults LB Mean	Adults LB 95P	Adults MB Mean	Adults MB 95P	Adults UB Mean	Adults UB 95P
Quinoline Yellow (E 104)	0.000	0.001	0.003	0.009	0.006	0.018
Allura Red AC (E 129)	0.058	0.029	0.007	0.030	0.008	0.030
Brown HT (E 155)	0.000	0.000	0.003	0.005	0.006	0.011
Steviol glycosides (E 960 a-d)*	0.130	0.667	0.133	0.667	0.136	0.669
mRPI	0.14	0.70	0.15	0.71	0.16	0.73

*expressed as steviol equivalents

Table S9. Detailed overview of Reference Point Quotient of the food additives and its modified Reference Point Index (mRPI) for the sensitive endpoint development toxicity

Food additive	Adults LB Mean	Adults LB 95P	Adults MB Mean	Adults MB 95P	Adults UB Mean	Adults UB 95P
Curcumin (E 100)	0.153	0.764	0.159	0.765	0.166	0.770
Quinoline Yellow (E 104)	0.001	0.005	0.015	0.047	0.032	0.091
Ponceau (E 124)	0.001	0.006	0.003	0.009	0.004	0.013
Allura Red AC (E 129)	0.006	0.029	0.007	0.030	0.008	0.030
Sorbic acid and its potassium salt (E 200-202)	0.137	0.401	0.144	0.408	0.152	0.420
Benzoic acid and sodium/potassium benzoate (E 210-213)	0.019	0.092	0.025	0.096	0.030	0.104

Glutamic acid/ glutamates (E 620-625)	11.66	44.84	11.67	44.86	11.68	44.87
Aspartame (E 951)	0.001	0.006	0.003	0.009	0.005	0.012
Steviol glycosides (E 960 a-d)*	1.603	9.820	1.605	9.820	1.608	9.821
mRPI	**13.6**	**56**	**13.6**	**56**	**13.7**	**56.1**

*expressed as steviol equivalents

Table S10. Detailed overview of Reference Point Quotient of the food additives and its modified Reference Point Index (mRPI) for the sensitive endpoint development toxicity revised exposure data

Food additive	Adults LB Mean	Adults LB 95P	Adults MB Mean	Adults MB 95P	Adults UB Mean	Adults UB 95P
Curcumin (E 100)	0.153	0.764	0.159	0.765	0.166	0.770
Quinoline Yellow (E 104)	0.001	0.005	0.015	0.047	0.032	0.091
Ponceau (E 124)	0.001	0.006	0.003	0.009	0.004	0.013
Allura Red AC (E 129)	0.006	0.029	0.007	0.030	0.008	0.030
Sorbic acid and its potassium salt (E 200-202)	0.137	0.401	0.144	0.408	0.152	0.420
Benzoic acid and sodium/potassium benzoate (E 210-213)	0.019	0.092	0.025	0.096	0.030	0.104
Glutamic acid/glutamates (E 620-625)	0.430	1.012	0.436	1.019	0.442	1.026
Aspartame (E 951)	0.001	0.006	0.003	0.009	0.005	0.012
Steviol glycosides (E 960 a-d)*	0.132	0.675	0.135	0.676	0.137	0.677
mRPI	0.9	**3**	0.9	**3.1**	1	**3.1**

*expressed as steviol equivalents
